# A self-management app to improve asthma control in adults with limited health literacy: a mixed-method feasibility study

**DOI:** 10.1186/s12911-023-02300-6

**Published:** 2023-09-27

**Authors:** Hani Salim, Ai Theng Cheong, Sazlina Sharif-Ghazali, Ping Yein Lee, Poh Ying Lim, Ee Ming Khoo, Norita Hussein, Noor Harzana Harrun, Bee Kiau Ho, Hilary Pinnock, RESPIRE Collaboration

**Affiliations:** 1 Department of Family Medicine, Faculty of Medicine and Health Sciences, Universiti Putra Malaysia, Serdang, Malaysia; 2 Malaysian Research Institute On Ageing, Universiti Putra Malaysia, Serdang, Malaysia; 3 UM eHealth Unit, Faculty of Medicine, Universiti Malaya, Petaling Jaya, Malaysia; 4 Department of Community Health, Faculty of Medicine and Health Sciences, Universiti Putra Malaysia, Serdang, Malaysia; 5 Department of Primary Care Medicine, Faculty of Medicine, Universiti Malaya, Kuala Lumpur, Malaysia; 6 Klinik Kesihatan Pandamaran, Ministry of Health Malaysia, Klang, Selangor Malaysia; 7 Klinik Kesihatan Bandar Botanik, Ministry of Health Malaysia, Klang, Selangor Malaysia; 8 NIHR Global Health Research Unit On Respiratory Health (RESPIRE), Usher Institute, The University of Edinburgh, Edinburgh, UK

**Keywords:** Mobile application, Asthma, Self-management, Health literacy, Feasibility study, Low-and-middle-income countries (LMIC)

## Abstract

**Background:**

Digital technology tailored for those with limited health literacy has the potential to reduce health inequalities. Although mobile apps can support self-management in chronic diseases, there is little evidence that this approach applies to people with limited health literacy. We aimed to determine the acceptability of a self-management app in adults living with asthma and have limited health literacy and the feasibility of delivering the intervention and assessing outcomes.

**Methods:**

We recruited eligible adults from the Klang Asthma Cohort registry in primary care for a 3-month mixed-method study plus a 2-month extended observation. We collected baseline data on socio-demography, health literacy and asthma control level. The outcomes of the intervention were assessed at 1- and 3-month: i) adoption (app download and usage), ii) adherence (app usage), iii) retention (app usage in the observation period), iv) health outcomes (e.g., severe asthma attacks) and v) process outcomes (e.g., ownership and use of action plans). At 1-month, participants were purposively sampled for in-depth interviews, which were audio-recorded, transcribed verbatim, and analysed deductively.

**Results:**

We recruited 48 participants; 35 participants (23 Female; median age = 43 years; median HLS score = 28) completed the 3 months study. Of these, 14 participants (10 Female; median age = 48 years; median HLS score = 28) provided interviews. Thirty-seven (77%) participants adopted the app (downloaded and used it in the first month of the study). The main factor reported as influencing adoption was the ease of using the app. A total of 950 app usage were captured during the 3-month feasibility study. App usage increased gradually, peaking at month 2 (355 total log-ins) accounting for 78% of users. In month 5, 51.4% of the participants used the app at least once. The main factors influencing continued use included adherence features (e.g., prompts and reminders), familiarity with app function and support from family members.

**Conclusions:**

An asthma self-management app intervention was acceptable for adults with limited health literacy and it was feasible to collect the desired outcomes at different time points during the study. A future trial is warranted to estimate the clinical and cost-effectiveness of the intervention and to explore implementation strategies.

**Supplementary Information:**

The online version contains supplementary material available at 10.1186/s12911-023-02300-6.

## Background

Asthma is a chronic inflammatory airway disease affecting an estimated 360 million people worldwide [1–3], yet it is a neglected chronic disease in many health settings [4]. In Malaysia, the prevalence of adult asthma was 4.2%, with 1.2% of deaths related to asthma in 2006 [5]. The hallmark of asthma is variability of symptoms and severity highlighting the importance of self-management, as a patient needs to be able to recognise when their condition is worsening and take remedial actions.

Health literacy is defined as an individual's cognitive and functional ability to navigate healthcare and respond to the demands of caring for their health [6]. Limited health literacy reduces people's ability to manage chronic conditions like asthma on their own [6, 7]. This is a global health issue, particularly in low-middle-income countries (LMICs), and has been shown to lead to poorer health outcomes [8] in conditions such as diabetes [9] and heart problems [10] although the relationship with asthma outcomes is unclear [7, 11].

Digital technology enables features to be tailored for those with limited health literacy by using creative illustrations and innovative video-based education as an alternative to wordy instructions, thereby potentially reducing health inequalities. Previous trials have shown that mobile apps can have a positive impact on asthma control and medication adherence [12, 13], for example supporting self-management and behaviour change in young people with asthma [14]. Nevertheless, none had looked at the acceptability and impact on populations with limited health literacy [15].

Given the global importance of the issue [15], developing and evaluating an intervention to improve asthma self-management tailored for people with limited health literacy is thus timely. Aligned with national and international recommendations for quality asthma care [16, 17], we used the Design Sprint methodology and incorporated results from our preliminary qualitative studies [18–20] and the advice of stakeholders [21] to develop a prototype app that includes information about asthma, medication and appointment reminders, an accessible pictorial asthma action plans and sources of social support [21]. In this study, we aimed to assess the acceptability of the app to adults with limited health literacy living with asthma and the feasibility of delivering the intervention and assessing relevant outcomes.

## Methods

Ethical approvals were obtained from the National Medical Research Ethics Committee, Ministry of Health, Malaysia [NMRR-18–2683-43494], Liverpool School of Tropical Medicine (LSTM) Research Ethics Committee (20–025) and relevant authorities involved in the Klang District. This study also received sponsorship approval from the Academic and Clinical Central Office for Research & Development (ACCORD) at the University of Edinburgh. Informed consent was obtained from eligible participants from the outset. Our feasibility study used a mixed-method approach to develop an understanding of how patients with limited health literacy would adopt and use an app to support asthma self-management.

### Study design and setting

**Fig. 1:**
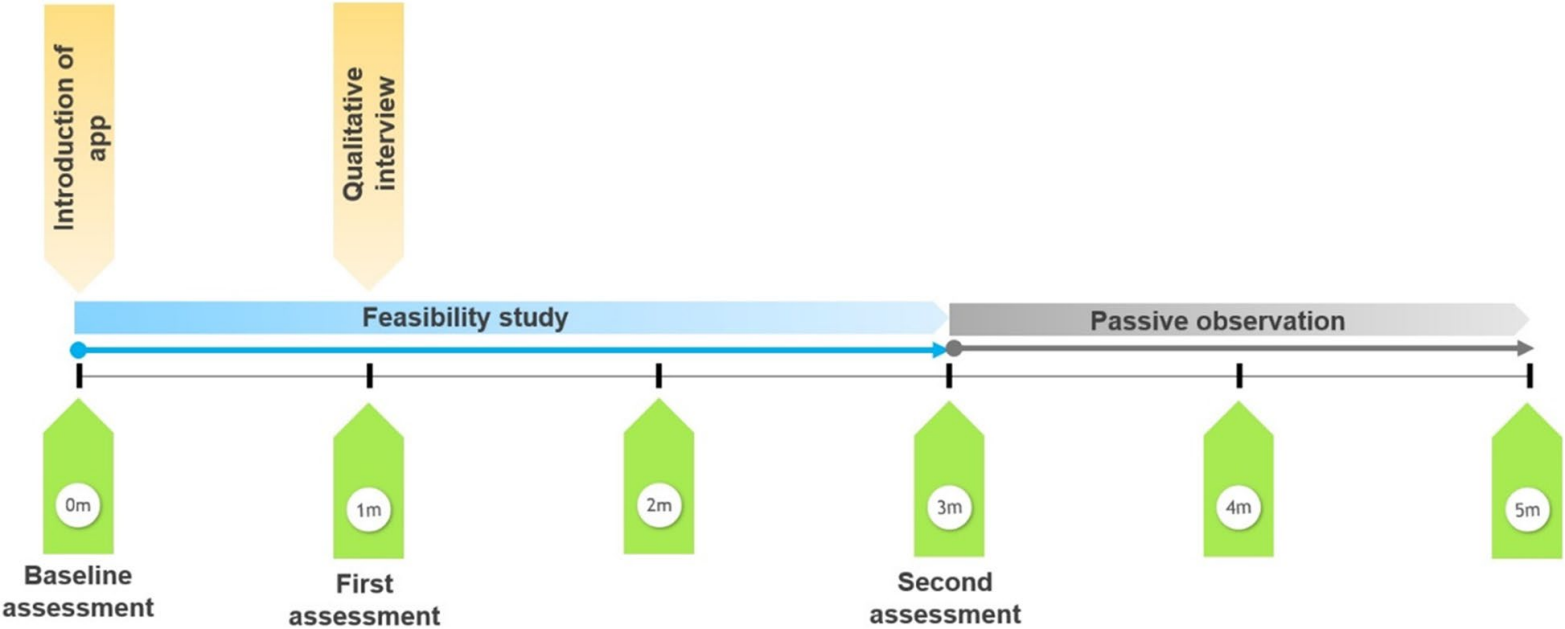
Outline of the study phases

Embedded within the Medical Research Council framework for the design and evaluation of complex interventions [22, 23], we conducted a three-month mixed-methods feasibility study plus two months observation of continued usage at three primary care clinics in Selangor, Malaysia between April and December 2021. We used GRAMMS to ensure complete and transparent reporting [24]. Figure 1 outlines the phases of the study. The mixed urban/rural state of Selangor was chosen as it has a high prevalence of adults with asthma (22%) [5] as well as the highest prevalence of limited health literacy in Malaysia at 75% [7, 25]. The selected clinics had a cohort of asthma patients registered in the Klang Asthma Cohort [26, 27].

### The context of asthma care in the study setting

Supplementary file 1 provides an overview of Malaysia’s healthcare system and sociodemographic context. Below is an outline of usual asthma care services provided by the participating primary care clinics in Klang, Selangor and a description of the intervention.

#### Usual care

We recruited patients from three primary care clinics. One clinic has a dedicated asthma clinic that operates one afternoon per week, while the other two clinics review patients with asthma in the general outpatient pools. In all these clinics, care is provided by a team including medical officers, pharmacists and nurses. Medical officers are doctors without formal postgraduate training who work in primary care under the supervision of specialist family physicians. The doctors are trained to manage asthma; pharmacists demonstrate inhaler techniques and discuss medication adherence and asthma action plans, while the nurses provide asthma education. For participants in the feasibility study, this routine multidisciplinary clinic management continued as usual throughout the study, but with the addition of the digital intervention.

### Intervention

Table 1 summarises the app’s key features developed with various stakeholders (healthcare professionals, people living with asthma and have limited health literacy) as described in our previous publication [21]. The intervention is a mobile app tailored to health literacy needs in which the app’s features address four aspects of care: (i) education; (ii) supporting self-management; (iii) supporting behaviour change; and (iv) social support. Supplementary file 2 illustrates the interfaces of the app and an outline of the app’s key features. The app was developed in *Bahasa Malaysia,* the national language of Malaysia.

**Table 1:** Key features of the MenangiAsmaAnda (WinMyAsthma) app

The aspect of asthma care	Key features
Education	About asthma
	Asthma medications
Supporting self-management	Self-monitoring of symptoms
	Asthma action plan
Supporting behaviour change	Asthma medication and appointment reminder
	Asthma calendar
	Reward system
Others	Social support

### Outcomes for assessing acceptability and feasibility

The outcomes of the intervention that were assessed at 1- and 3-month intervals are as follows:

i) Adoption (app download and usage),
ii) Adherence (app usage),
iii) Retention (app usage in the observation period),
iv) Health outcomes (e.g., severe asthma attacks) and.
v) Process outcomes (e.g., ownership and use of action plans).

We chose the definition of acceptability components (adoption, adherence and retention of tasks) based on the Fogg Behaviour grid [28]. This is a model of persuasive technology that identifies those features that the technology needs to include to change users’ behaviours from how people generally perform [28] (see Table 2 for operational definitions).

**Table 2:** The types of data collection methods and analysis

Data	Components or operational definition	Instrument	Data analysis
Adoption	The number of people downloading the intervention and starting to use the app (at least once)	Google Analytics	• The number of participants who download the app
Adherence	Continued use of an app or telehealth, not necessarily every day but at least once a month throughout the 3 months	App usage data	• The number of apps use within the 3-month feasibility study
Retention	The number of people who continued using the app in the observational phase (months 4 and 5)	App usage data	• The number of participants who used the app during the 2-month observation period • The number of apps use during the 2-month observation period
Sociodemographic	Information on age, gender, ethnicity, level of education and household income	Questionnaire	• A descriptive summary of the baseline sociodemographic characteristics and outcomes were reported using median and interquartile range for continuous variables and frequencies and percentages for categorical data
	Health literacy level was measured using a validated 47-item questionnaire	Health Literacy Scale (HLS) [6, 29]	• Limited health literacy is defined as an Index of $<$ 33 points [29] and was reported using median and interquartile range
Health outcome			
• Asthma control	Asthma control was measured using a 4-item validated questionnaire	Global INitiative for Asthma (GINA) Asthma Symptoms Control tool [30]	• A descriptive summary of the relevant outcomes was reported using median and interquartile range for continuous variables and frequencies and percentages for categorical data
• Severe asthma attacks	Deterioration of asthma control that required urgent action on the part of the patient and physician to prevent a serious outcome, such as hospitalisation or death from asthma [31] Relevant actions included commencing a course of oral steroids, inhaler use through aerochamber (during the COVID-19 pandemic) and emergency department visit for nebulisation or hospitalisation	Questionnaire	
Process outcome	Ownership of asthma action plan, use of asthma action plan with the app and attendance to follow-up	Questionnaire	
Facilitators and challenges in using the app	Exploring the app's use facilitators and challenges, with a focus on the potential factors influencing app adoption, adherence, and retention	Interviews	• Interviews were facilitated using a topic guide based on the Fogg Behaviour grid [28]
			• Interviews were audio-recorded, transcribed verbatim, and analysed deductively

We assessed the feasibility of collecting health and process outcomes at 1- and 3-month intervals. Health outcomes include asthma control, number of severe attacks, number of steroid courses, number of emergency visits and hospitalisations (see Table 2 for operational definitions). Process outcomes include ownership of an asthma action plan, use of action plan, and attendance at follow-up.

### Recruitment and enrolment

#### Klang asthma cohort

Patients were recruited from the Klang Asthma Cohort (NMRR-18–2707-42719) [26, 27]. The registry is a research output of the NIHR Respiratory Global Health Unit (RESPIRE) in Malaysia [26, 27]. The database contains the clinical and demographic data of a cohort of people with asthma who have been recruited from primary healthcare clinics in the Klang district and who have consented to be contacted with invitations to participate in research. The database also includes demographic information e.g., age, ethnicity and medical information, spirometry results and medication lists.

#### Approaching potential participants

Potential participants were purposively sampled from the Klang Asthma Cohort database based on age, gender and ethnicity to reflect a broad range of socio-demographic characteristics [26, 27]. Those who fulfilled the inclusion and exclusion criteria were then invited to take part (see Table 3). They were contacted via a telephone call by a trained research assistant who provided a detailed description of the study.

**Table 3:** Eligibility criteria

Inclusion criteria	Exclusion criteria
• Patients with physician-diagnosed asthma as recorded in the medical records	• Any person who is acutely unwell and requires emergency management at the time of the consultation
• Aged 18 years and above (based on the Malaysian Age of Majority Act 1971 that allows them to make a medical decision on their own [32].)	• People unable to participate in interviews (e.g., severe hearing/speech impairment) in the most widely used languages in Malaysia (*Bahasa Malaysia*, English)
• Patients who use an inhaled corticosteroid (preventer inhaler) daily	• Unable to give informed consent (e.g., cognitive impairment, learning disability)
• People with limited health literacy (defined as an index of < 33 on the validated health literacy scale (HLS-Asia Q47))	• At the discretion of the doctor because of overriding medical conditions or social issues (e.g., terminal illness, recent very distressing life event)
• Patients who are android phone users (the mobile operating system we used for our prototype)	

Potential participants were given two weeks to decide on participation. The research assistant then arranged a meeting for those who agreed to participate either face-to-face or remotely depending on the COVID-19 movement restrictions in force at the time. All meetings were conducted remotely and at the meeting, the study process was explained by a researcher, and written or verbal informed consent was obtained. Baseline data were collected, and a short training session using a video-based training module about the app was conducted for every participant which included instructions and content on how to download and use the app.

### Sample size

As this is a feasibility study, we did not perform a sample size calculation. We planned to approach 60 eligible participants from the Klang Asthma Cohort with the aim of recruiting approximately 35 participants to use the app. This number is adequate to inform us about the feasibility of delivering a mobile app for asthma self-management intervention, as well as to assess the recruitment process and attrition [33].

### Data collection

Table 2 shows the types of quantitative and qualitative data collected in this study. Quantitative data were collected (in *Bahasa Malaysia* or English languages) using a pre-tested structured questionnaire for sociodemographic information and validated tools for asthma control. Adoption, adherence and retention were assessed by observing app usage of symptom-monitoring and asthma action plan interfaces, which also captured asthma control (see Table 2).

Information on health and process outcomes were self-reported and verified by clinic doctors from the participant's medical records. We used self-reported methods as participants in Malaysia have access to private healthcare facilities so all events may not be captured by the public healthcare facilities. Follow-up data on all the outcomes were collected at baseline, 1- and 3-month post-intervention via telephone calls by trained enumerators.

For the qualitative interviews, we purposively sampled participants for in-depth interviews. All the interviews were conducted remotely (by PYL, CAT, SSG or HS) in line with the COVID-19 movement restriction order at the time. A topic guide was used to facilitate the interview (Supplementary file 3) [28].

### Data analysis

Table 2 outlines the analysis of the different types of data. The SPSS statistics v27 [34] and Nvivo 11 software [35] were used to conduct these analyses.

The study was not powered to demonstrate effectiveness. To prevent over-interpretation, we have undertaken any statistical analysis of the health outcomes and have not presented the results within the main paper. However, Supplementary file 4 (Table 1) makes available the raw quantitative data for potential use in a future meta-analysis.

Qualitative data were analysed iteratively throughout the data collection process and were informed by, but not restricted to, the domains of the Fogg Behaviour grid [28]. HS, PYL and CAT deductively coded the transcripts using the components concerning apps use; adoption, adherence and retention. Each interview statement was coded into one of these components. Refinement and agreement of the analysis process were done in iterative discussion with the multidisciplinary research team.

## Results

**Fig. 2:**
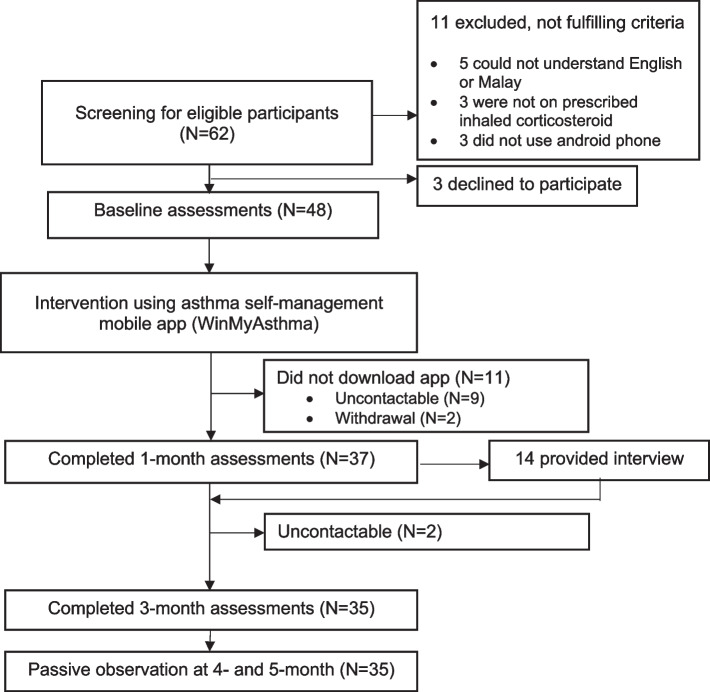
Flow of study participants

Figure 2 summarises the flow of the participants in the study. We randomly screened the records of 62 adult patients with asthma from the Klang Asthma Cohort registry between April to July 2021, identifying and inviting 51 potential participants of whom 48 attended the initial baseline assessment.

### Baseline characteristics of participants

The asthma self-management mobile app was introduced to 48 participants. Table 4 provides the baseline descriptive data of these participants. After the introduction to the app, 11 out of 48 participants were found to not download the app; nine participants were uncontactable after three attempts, and none provided any reason for not opting to try the app. Two others experienced mobile phone issues (either broken or lost phones), and thus officially withdrew. They were mainly women (8/11, 73%), of Malay ethnicity (5/11, 45.5%), with a secondary level of education (6/11, 54.5%) but relatively older with a median age of 55 (26) as compared to those who stayed on the study. The median (IQR) household income was USD 560 (447) and the health literacy score was 28 (4).

**Table 4:** Baseline characteristics of the study participants at the different study stages

		Enrolled participants, *N* = 48		Feasibility study (3-month), *N* = 35		Interview, *N* = 14	
Variables		n	%	n	%	n	%
Gender	Male	15	31.3	12	34.3	4	28.6
	Female	33	68.8	23	65.7	10	71.4
Ethnicity	Malay	23	47.9	15	42.8	6	42.9
	Indian	15	31.3	10	28.6	4	28.6
	Chinese	9	18.8	9	25.7	4	28.6
	Others	1	2.1	1	2.9	0	0
Level of education	No formal education	2	4.2	1	2.9	0	0
	Primary level	7	14.6	4	11.4	1	7.1
	Secondary level	27	56.2	19	54.3	9	64.3
	Tertiary	12	25.0	11	31.4	4	28.7
Asthma control	Controlled	18	37.5	16	45.7	8	57.1
	Uncontrolled	30	62.5	19	54.3	6	42.9
Variables		Median (IQR)					
Age		46 (23)		43 (18)		48 (24)	
Household income, USD		448 (672)		582 (683)		560 (610)	
HLS score^a^		28 (5)		28 (6)		28 (7)	

Footnotes: Mean less than 33 = limited health literacy^a^; conversion rate: RM 1 = USD 0.22

For those who continued to take part in the 3-month feasibility study (*N* = 35), the median (IQR) age of the participants was 43 (18) years, 65.7% were women, 42.8% were Malay, 28.6% were Indians and 25.7% were Chinese and 2.9% were of other ethnicities. The median (IQR) household income was USD 582 (683), the health literacy score was 28 (6) and 45.7% had controlled asthma.

### Acceptability of app intervention

#### Adoption

Out of 48 eligible participants, 77.1% (*n* = 37) downloaded the app, all of whom logged into the app at least once in the first month of the feasibility study.

From the qualitative interviews, the participants described ease of use as the main reason that encouraged them to download and start using the app. The app was easy to use because of the readability of its fonts, and clear navigation icons. As a participant recalled,

*No, no. No problem with the log-ins. Not that I can recall. […] That one [app] is quite easy to move from one page to another; got [an] arrow and all. [The] functions very simple to know what they're for. The images all are straightforward. One look I know what it is.*

*67-year-old Chinese man, with well-controlled asthma*

’Assisting research’ was one of the main reasons for downloading and using the app.

*I think I like it when research takes into account patients' views. When the RA (research assistant) told me that after I used the app, one of you will interview me to ask about this app. So, I agree. I want to help the research and be more useful to the research, making the app better.*

*46-year-old Malay woman, with well-controlled asthma*

Other motivations included a desire to learn more about asthma, a willingness to manage their health, and believing the app could support future meetings with healthcare professionals.

Most participants made recommendations on features of the app that they considered could promote adoption. Such features include a health or emergency services finder (help to find facilities nearest to user location), instant feedback or live chat option with healthcare professionals, healthy lifestyle advice such as exercise and food and environment such as weather information.

*Information about good nutrition, or the weather information in the app. I tend to have an [asthma] attack when I'm cold and it rained heavily. So I don't leave the house much, right? Or like haze. So if I need to go out, I‘ll bring the right mask. Real-time information, you see. If you can add, these are my suggestions and I’m sure people will want to use them. I will!*

*42-year-old Indian man, with uncontrolled asthma*

#### Adherence

**Fig. 3:**
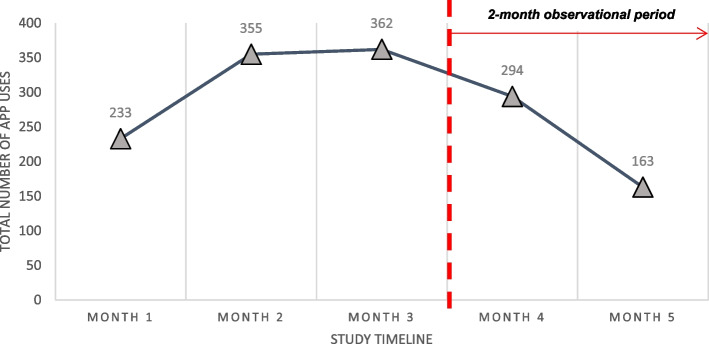
Graphical representation of the total number of app uses during and after the feasibility study

Figure 3 illustrates the total app usage during and after the feasibility study phase. A total of 233 usages were detected in the first month of the feasibility study which accounts for 24.5% (233/950) of the total log-ins during the 3 months of the active intervention period.

**Fig. 4:**
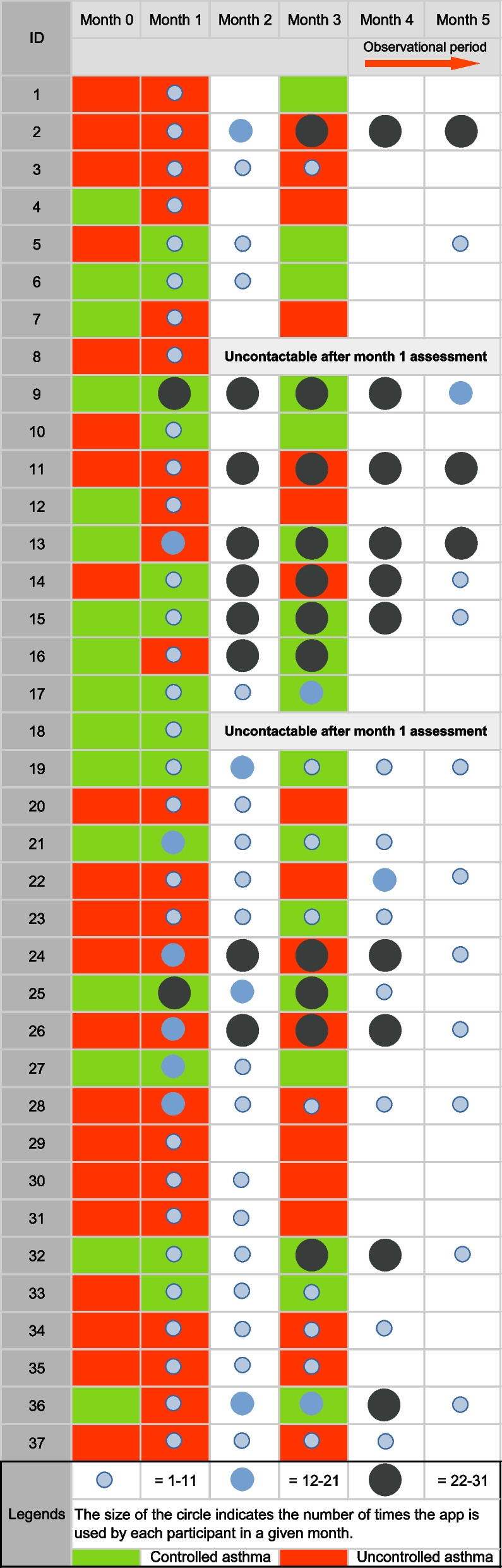
App usage data throughout the study period, *N* = 37

Adherence increased over time within the 3-month feasibility study. A total of 950 app uses were captured during the 3-month feasibility study and a 52.4% increase in app usage was detected in the first 2 months (see Fig. 3). This is despite eight participants not using the app after the first month (see Fig. 4). App usage plateaued over the second and third months of the active intervention period.

Figure 4 provides a graphical representation of the adherence pattern for individual participants. More than two-thirds of the participants (78.1%; 29/37) continued using the app for 2-months and 59.5% (22/37) continued using the app throughout the 3 months. Supplementary file 5 provides detailed information on app usage for each participant during the 3-month feasibility study.

From the qualitative interviews, participants described that prompts for daily symptom-monitoring and medication use promoted daily usage. Daily use improved familiarisation with the app, and to some extent, the ability to memorise the steps of the action plan.

*I occasionally browsed apps on my phone including this [asthma] app and I tend to use it when I need to - it's accessible. The good thing is, when I always look at the [asthma action] plan, I seemed to remember what steps to take. Now I think I know what to do. I don't have to look at this app if I suddenly feel short of breath. It is like that lah (a colloquial expression to emphasise a statement).*

*28-year-old Malay woman, with well-controlled asthma*

Participants identified poor internet accessibility and quality as barriers to continued use. One frustrated participant described his internet quality,

*At times where there’s inter...internet interruption. Like no line. I was recording my medication intake, but [I] cannot key it in, you see. It showed ‘loading’, ‘loading’, ‘loading’ and then...and the app didn’t record what I put in. Very frustrating and this happened [a] few times. […] This area sometimes [has] bad internet connection.*

*64-year-old Chinese woman, with uncontrolled asthma*

Older participants, who initially perceived that they may not be able to engage with technology, received help from younger members of the family who were living with them. Such support served as a motivation and an opportunity to continue using the app independently, as reported by a participant,

*My children, they look [at the app] together with me at first. Initially, I don’t think at my age I can use this [app]. They taught me how to use; this medication, that medication. So, it helps me to understand how to use this [app] on my own.*

*51-year-old Malay woman, with uncontrolled asthma*

#### Retention

The app was used 457 times by the study participants in the 2-month post-study observation period (see Fig. 3). Slightly more than half (19/37, 51.4%) of the study participants continued using the app at least once in months 4 and 5 and more than a third (13/37, 35.1%) used the app more than once a month for the two months after the 3-month study had finished (see Fig. 4). We did not interview participants after three months of app use. However, one participant who we observed using the app after the study period ended (see Fig. 4: ID 9) expressed her desire to continue using the app after the study ended despite her initial struggle with log-ins. During the 1-month qualitative interview, she said,

*I forgot [the] password [after] I logged out. But, you (the researcher) give me [the password] again. I [did] not realise that there is a ‘forget password’ button [in the app]. […] But despite this, [I] will continue using this [app]. Very useful and I don't think it takes much time to use. Is this possible – when [the] study finish (ended)?*

*31-year-old Indian woman with well-controlled asthma*

#### Assessing health and process outcomes

It proved feasible to assess the outcomes of interest at baseline, 1-month and 3-month time points remotely. At baseline, 62.5% (30/48) of the participants were classified as ‘uncontrolled’, and 20.8% (10/48) had had a severe attack in the previous 3 months. At 3 months 48.6% (17/35) of the participants were classified as ‘uncontrolled’, and 5.7% (2/35) had had a severe attack in the previous 3 months. There were no adverse events during the 3-month feasibility study.

The study is not powered to demonstrate a significant effect on these outcomes; thus, we did not present the results within the main paper. However, we provided Supplementary file 4, containing the detailed quantitative data we collected, for further reading.

## Discussion

### Statement of principal findings

The dual aims were to assess the app's acceptability and the feasibility of delivering the intervention and collecting key outcomes for determining processes in a future evaluation. Adoption rates were high, with 77% of eligible participants downloading the app and using it within the first month. Adherence peaked in the second month (78% of users), and more than half (51.4%) continued using the app for two additional months. Factors influencing continued use included app adherence features, improved familiarity through daily use, and family support. The data collection point at the month-1 review potentially facilitated interest in the app. Moreover, the study successfully collected proposed health and process outcomes at various time points throughout the study phases.

### Strengths and limitations

Our study has several strengths and limitations. This study provides a novel insight into the acceptability and feasibility of delivering a mobile app for people with limited health literacy to support asthma self-management, thus providing useful information for future evaluation of effectiveness and implementation. The mixed-methods approach enabled us to synthesise both quantitative and qualitative data, providing a better understanding of the research findings. Our participants may not have been representative of the broader population with asthma in Malaysia. They were recruited from the RESPIRE’s Klang Asthma Cohort (KAC) [26, 27], so were likely to already be interested in regular care and/or supporting research in asthma management. Having an Android smartphone was another prerequisite for participation (android is the largest mobile operating system in Malaysia [36]); therefore, our participants were potentially a group of people who are technology savvy and interested in technology. The inclusion of those who owned phones running the iOS operating system may have increased participation, but developing and supporting an iOS system was beyond our budget at this feasibility piloting stage. The participating healthcare centres are in the urban setting and may serve people who have better internet access than in other parts of Malaysia, thus reducing the generalisability of our findings to other parts of the country or LMICs with less-developed internet infrastructures. Despite limited health literacy level, based on our screening, participants were able to complete the study with minimal support which we also found from our previous findings [21]. Five participants were excluded as they were not able to speak English and/or *Bahasa Malaysia*, which means that the findings may not apply to those who solely speak and understand Mandarin and Tamil languages. The participant recruitment started at different times of the year affected by the changing COVID-19 movements restriction order (e.g. school closure required parent participants to home-school children) or holiday periods (e.g. Ramadhan) which may have affected the app adoption rate. At the beginning of the study, eleven participants did not download the app, nine of whom were uncontactable; thus we were not able to explore the reasons for not downloading the app. However, we do know that they were older compared to those who stayed in the study. As the app prototype is not sophisticated, we were unable to extract detailed engagement of each feature of the app e.g. time spent on each interface and the number of times participants returned to the asthma action plan without the symptoms-monitoring prompts. Finally, the decision not to undertake statistical analysis of the health outcomes reflects the role of this feasibility study to explore the processes of delivering and evaluating the intervention and the limitation imposed by the small sample size. A future fully powered assessment will be required to determine the effectiveness (or not) of the refined intervention. However, there was no evidence of any harm. To assess the potential harm of using the intervention, particularly on health, we conducted scheduled phone calls and explored any potential adverse health events during the qualitative interviews with the participants. Additionally, participants were actively encouraged to report any adverse events they experienced to the research team while using the mobile app for asthma management. This proactive approach to identifying any adverse events potentially related to the app's usage enabled us to ensure the safety of the participants throughout the study and also to raise any potential concerns for monitoring in a future fully powered evaluation.

### Comparison with prior work

Mobile applications (apps) are widely used for health management owing to their flexibility and capacity to personalise components to care for health [21, 37]. The digitalisation of healthcare may provide cost-effective, accessible care [38, 39], but it may also risk creating a digital divide if such innovation excludes marginalised and vulnerable populations who are most in need of care, such as people with limited health literacy [40, 41]. Among the described barriers to accessing and using health technologies are issues with the readability of content and usability [41], both of which our prototype addressed [21]. Despite the concern that technology may increase health inequality [42], we have evidence to suggest that an app tailored to health literacy needs is acceptable among people with limited health literacy and that it was feasible to assess important health and process outcomes at different time-points for a future trial.

In terms of acceptability, we measured adoption by the number of participants who downloaded the app and from the interviews, we identified several factors that promoted adoption. One of these was a desire to assist research; though this motive is not applicable in real life. Other motivators for app download included ease of download and family members' support for older participants. Ease of app use and features such as low cost and portability have been reported as being important in low-resource settings as these features can support the cost-effective scalability of digital solutions [43]. Family involvement in interventions is commonly reported in conditions involving children [44] and mental disorders [45] but not highlighted in asthma. Family involvement has shown promising results, including improved uptake of self-management tasks and health outcomes [46, 47], highlighting the role of family in creating favourable illness experiences. Despite the high adoption rate, eleven people did not download the app despite training. While an app is a novel way to achieve a proven end (supported self-management), which may engage different people, it does not have to be the only way of delivering care. Face-to-face communication may suit other people better. Other practical factors in this group of people include deteriorating near vision, as the content in a mobile app can be small and requires good acuity.

In our study, adherence increased over time. We found that symptom-monitoring features and daily preventer reminders were the ‘adherence features’ described by participants for supporting the continued use of the app. These preferred features by the participants in our study are consistent with findings from Hui et al., in which participants described the need for instant feedback on the monitoring [48], similar to suggestions from our participants. The increasing adherence in our study was potentially attributed to a better understanding of the app's functions with familiarity, and it was possible that the 1-month evaluation and interviews enhanced usage. Discussions about the app's potential uses and benefits may have triggered participants' interest in exploring it, and with some basic skills learned in the previous month of use, they were ready to discover previously unused features within the app. On a practical level, a 1-month contact to discuss the app could be a useful implementation strategy in a future trial.

Poor internet accessibility deters continued use among participants in our study. Studies have shown that those with poor or limited access to the internet or a reliable power supply may not be able to engage with technology effectively [49], thus, there is a need for a national digital health policy in line with efforts to improve healthcare delivery in low-resource settings. Future studies should explore the policy, system and population context-specific readiness for digital health transformation in LMICs.

We noted that although several key participants continued to use the app after the study ended, app use wanes over time which is consistent with other studies that reported a similar trend in the level of user engagement with digital health technology [50–52]. Participants reported being better at managing asthma symptoms without assistance, and the drop in app usage rates may reflect a successful learning process, rather than a failure of retention. Although technology may not be for everyone, the continued use of the app after the study ends suggests that digital intervention has a role in supporting asthma care, especially in technology users and those who are motivated to change behaviour [43].

## Conclusion

In conclusion, an asthma self-management app to improve asthma control in adults with limited health literacy was acceptable for adults with asthma and it was feasible to collect desired outcomes at different time points during the study. Digital solutions have the potential to bridge the gap in health inequality through inclusive development and implementation. A future trial is warranted to estimate the clinical and cost-effectiveness of the app intervention, explore implementation strategies and establish evidence-based asthma apps for people with limited health literacy.

### Supplementary Information

**Additional file 1: Supplementary file 1. **Malaysia: its health system and social context.

**Additional file 2: Supplementary file 2. **i) Examples of the app interfaces. ii) The details of the app’s features.

**Additional file 3. **

**Additional file 4: Supplementary file 4 Table 1. **Summary of the quantitative data collected throughout the 3-month feasibility study. **Table 2.** The potential effect of mobile app intervention on health and process outcome measures. 

**Additional file 5. **

## Data Availability

The datasets used and/or analyzed during the current study are available from the corresponding author on reasonable request. The dataset that supports the conclusions is available within the manuscript.

## Abbreviations

AAP: Asthma action plan
HLS: Health literacy scale
KAC: Klang Asthma Cohort
LMIC: Low-and-middle-income countries
MRC: Medical Research Council
NIHR: National Institute of Health and Care Research
RESPIRE: NIHR Respiratory Global Health Unit
