# Supplementary material for: A self-management app to improve asthma control in adults with limited health literacy: a mixed-method feasibility study
Source: BMC Med Inform Decis Mak. 2023 Sep 27;23:194. doi: 10.1186/s12911-023-02300-6 (PMC10523795; doi:10.1186/s12911-023-02300-6)
Supplement: Supplementary file 1 — Additional file 1: Supplementary file 1. Malaysia: its health system and social context. [file 12911_2023_2300_MOESM1_ESM.docx]

# Supplementary file 1

Malaysia: its health system and social context.

| **The health system** | **Multiracial and multilingual Malaysia** |
| --- | --- |
| - Malaysia has both public and private healthcare systems. - The Government heavily funds the public health sector through taxation. The private sector is a ‘fee for service’ model often covered by insurance policies. - The Government provides primary, secondary and tertiary care for the population. National Referral Centres provide specialised care and support the primary care service [53]. - In the public health sector, services are free with co-payment ranging from MYR1 (GBP 0.17) to MYR 5 (GBP 0.87) for outpatient services and MYR 3 (GBP 0.54) per day of admission [54]. The maximum MYR 5 (GBP 0.87) fee is for outpatient specialist consultation [54]. | - Malaysia is a multiracial country comprising three main ethnic groups: Malays, Chinese and Indians, each with their own culture and language. |
|  | - The national language, Malay, is used as the main medium of instruction in both primary and secondary national schools. |
|  | - English is learned as a second language. |
|  | - At the primary school level, schools may also offer Mandarin and Tamil mediums of instruction. |
