# Supplementary material for: A self-management app to improve asthma control in adults with limited health literacy: a mixed-method feasibility study
Source: BMC Med Inform Decis Mak. 2023 Sep 27;23:194. doi: 10.1186/s12911-023-02300-6 (PMC10523795; doi:10.1186/s12911-023-02300-6)
Supplement: Supplementary file 2 — Additional file 2: Supplementary file 2. i) Examples of the app interfaces. ii) The details of the app’s features. [file 12911_2023_2300_MOESM2_ESM.docx]

# Supplementary file 2

i) Examples of the app interfaces.

| 1. ***Mainframe with medication reminders:***   **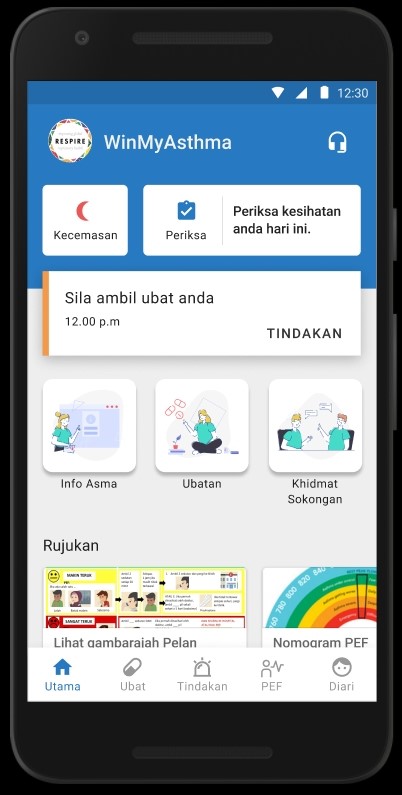** | 1. ***Medication listing:***   **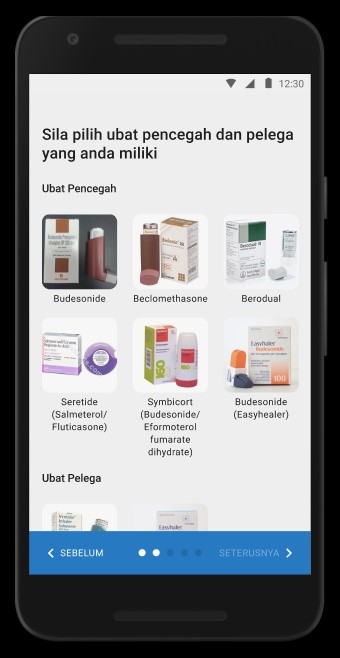** |
| --- | --- |
| 1. ***Information on PEFR:***   **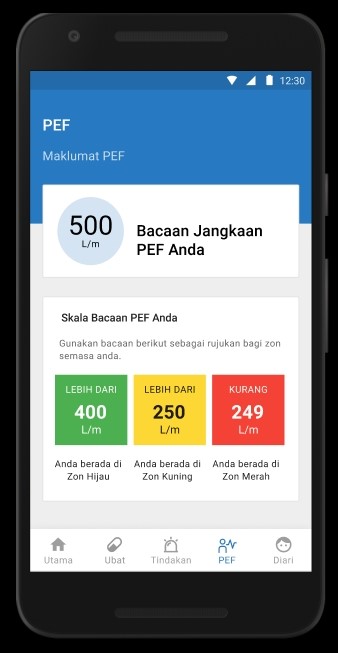** | 1. ***Pictorial asthma action plan:***   **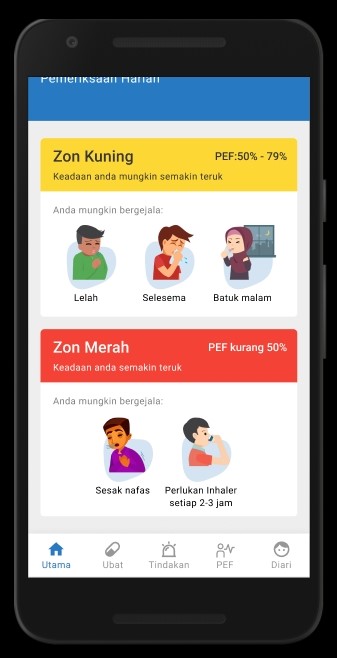** |

ii) The details of the app’s features

| Aspect of asthma care | Features | Content | Others |
| --- | --- | --- | --- |
| Education | About asthma | Information in text and videos about asthma, symptoms, triggers, how the diagnosis is made, exacerbations and myths around asthma attacks. Other information includes types of medications used to treat asthma, their function and potential side effects. There will be video-based instructions on inhaler technique. | The links to the Ministry of Health portal on asthma are provided under specific headings to facilitate the search for reliable information. |
|  | Asthma medications |  |  |
| Supporting self-management | Self-monitoring of symptoms | Patients indicate any experience of asthma symptoms in the last 24 hours, which will translate into an indication of asthma control level and prompt checking of the action plan. | Tick-box list of potential asthma symptoms; ticking any one symptom will prompt a pop-up on advice to look at an action plan with a click button. |
|  | Asthma action plan | Illustrations and wording were previously validated in discussions with stakeholders. | List of zones are displayed, and patient chooses which zone is appropriate for them. |
| Supporting behaviour change | Asthma medication and appointment reminder | Patients provide information about medications and follow-up consultations, which triggers reminders at the timing of choice. | Matrix of images of medications used and drop-down menu for frequency and timing. |
|  | Asthma calendar | Asthma control and medication uptake will be recorded in the diary, including best peak expiratory flow rate (PEFR). | Monthly calendar, which displays asthma control and adherence. |
|  | Reward system | Achieving good asthma control and medication adherence will be translated into points. | Display of scale of points achieved for good asthma control and adherence. |
| Others | Social support | Information regarding support groups for asthma in Malaysia. | The links to various support groups available in Malaysia. |
