# Supplementary material for: A self-management app to improve asthma control in adults with limited health literacy: a mixed-method feasibility study
Source: BMC Med Inform Decis Mak. 2023 Sep 27;23:194. doi: 10.1186/s12911-023-02300-6 (PMC10523795; doi:10.1186/s12911-023-02300-6)
Supplement: Supplementary file 3 — Additional file 3. [file 12911_2023_2300_MOESM3_ESM.docx]

# Supplementary file 3

| Components | Prompts |
| --- | --- |
| Trigger | - How would you describe your experience using the app so far? (registration, login) - What aspects of the app that you like or find challenging to use? - What would you like to see in an asthma app in the future? |
| Motivation | - What made you decide to try this application? - What will make you use the application for the next few months? |
| Ability | - How would this app help you to self-manage your asthma at home? - How would your surroundings help you to use the app? |
