# Supplementary material for: A self-management app to improve asthma control in adults with limited health literacy: a mixed-method feasibility study
Source: BMC Med Inform Decis Mak. 2023 Sep 27;23:194. doi: 10.1186/s12911-023-02300-6 (PMC10523795; doi:10.1186/s12911-023-02300-6)
Supplement: Supplementary file 4 — Additional file 4: Supplementary file 4 Table 1. Summary of the quantitative data collected throughout the 3-month feasibility study. Table 2. The potential effect of mobile app intervention on health and process outcome measures. [file 12911_2023_2300_MOESM4_ESM.docx]

# Supplementary file 4

Table 1: Summary of the quantitative data collected throughout the 3-month feasibility study.

|  | | Baseline  (0-month)  N=48 | | 1-month,  N=37 | | 3-month, N=35 | |
| --- | --- | --- | --- | --- | --- | --- | --- |
| Variable and timescale for assessment at baseline | | n | % | n | % | n | % |
| Asthma control *in the last 4 weeks* | Controlled | 13 | 27.1 | 14 | 37.8 | 17 | 48.6 |
|  | Uncontrolled | 35 | 72.9 | 23 | 62.2 | 18 | 51.4 |
| Number of severe attacks *in the last 6 months* | 0 | 38 | 79.2 | 36 | 97.3 | 33 | 94.3 |
|  | 1 | 2 | 4.2 | 0 | 0 | 1 | 2.9 |
|  | 2 | 3 | 6.3 | 1 | 2.7 | 0 | 0 |
|  | $\geq$3 | 5 | 10.4 | 0 | 0 | 1 | 2.9 |
| Number of steroid courses *in the last 6 months* | 0 | 38 | 79.2 | 32 | 86.5 | 28 | 81.1 |
|  | 1 | 7 | 14.5 | 4 | 10.8 | 3 | 8.1 |
|  | 2 | 3 | 6.3 | 1 | 2.7 | 4 | 10.8 |
|  | $\geq$3 | 0 | 0 | 0 | 0 | 0 | 0 |
| Number of emergency visits *in the last 6 months* | 0 | 45 | 93.8 | 36 | 97.3 | 33 | 94.3 |
|  | 1 | 0 | 0 | 0 | 0 | 0 | 0 |
|  | 2 | 1 | 2.1 | 1 | 2.7 | 2 | 5.7 |
|  | $\geq$3 | 1 | 2.1 | 0 | 0 | 0 | 0 |
| Number of hospitalisations *in the last 6 months* | 0 | 37 | 100.0 | 37 | 100.0 | 37 | 100.0 |
| Ownership of asthma action plan (AAP) *in the last 1 year* | No | 35 | 72.9 | 17 | 45.9 | 15 | 42.9 |
|  | Yes | 13 | 27.1 | 20 | 54.1 | 20 | 57.1 |
| Use of asthma action plan (AAP) *in the last 6 months* | No | 43 | 89.6 | 29 | 78.4 | 35 | 100 |
|  | Yes | 5 | 10.4 | 8 | 21.6 | 0 | 0 |
| Attendance to follow-up *in the last 6 months* | No | 5 | 10.4 | 3 | 8.1 | 7 | 14.3 |
|  | Yes | 43 | 89.6 | 34 | 91.9 | 30 | 85.7 |

Footnotes: For 1- and 3-month, the recall period is in the last 1 month and 3 months respectively.

Table 2: The potential effect of mobile app intervention on health and process outcome measures.

| Model Term | Unadjusted B | SE | Crude OR | 95%CI | | P value |
| --- | --- | --- | --- | --- | --- | --- |
|  |  |  |  | Lower | Upper |  |
| Asthma control^a^ |  |  |  |  |  |  |
| 0-month | Ref |  |  |  |  |  |
| 1-month | -0.225 | 0.4809 | 0.799 | 0.308 | 2.072 | 0.642 |
| 3-month | 0.218 | 0.4737 | 1.243 | 0.486 | 3.180 | 0.646 |
| Number of severe attacks |  |  |  |  |  |  |
| 0-month | Ref |  |  |  |  |  |
| 1-month | -0.622 | 0.2511 | - | -1.119 | -0.124 | 0.015* |
| 3-month | -0.459 | 0.2799 | - | -1.014 | 0.095 | 0.104 |
| Number of steroid courses |  |  |  |  |  |  |
| 0-month | Ref |  |  |  |  |  |
| 1-month | 3.749E-17 | 0.1027 | - | -0.204 | 0.204 | 1.000 |
| 3-month | 0.135 | 0.1307 | - | -0.124 | 0.394 | 0.304 |
| Number of emergency visits | | |  |  |  |  |
| 0-month | Ref |  |  |  |  |  |
| 1-month | -0.081 | 0.1103 | - | -0.300 | 0.138 | 0.464 |
| 3-month | -0.081 | 0.1033 | - | -0.286 | 0.124 | 0.434 |
| Ownership of asthma action plan (AAP) | |  |  |  |  |  |
| 0-month | Ref |  |  |  |  |  |
| 1-month | -0.896 | 0.4885 | 0.408 | 0.155 | 1.074 | 0.069 |
| 3-month | -1.006 | 0.4898 | 0.366 | 0.139 | 0.966 | 0.042* |
| Use of asthma action plan (AAP) | |  |  |  |  |  |
| 0-month | Ref |  |  |  |  |  |
| 1-month | -0.810 | 0.4948 | 0.445 | 0.167 | 1.186 | 0.105 |
| 3-month | 1.425 | 0.3490 | 4.156 | 2.081 | 8.300 | <0.001* |
| Attendance to follow-up |  |  |  |  |  |  |
| 0-month | Ref |  |  |  |  |  |
| 1-month | -5.795E-15 | 0.8635 | 1.000 | 0.181 | 5.538 | 1.000 |
| 3-month | -0.972 | 0.7442 | 0.378 | 0.086 | 1.653 | 0.194 |

** P<.05, B= coefficient, SE=Standard Error, CI: Confidence Interval, Ref=Reference group.*

*^a^Asthma control is defined as controlled and uncontrolled*

Footnotes:

- We undertook a mixed regression analysis with repeated measures (time 0^th^ -month, 1-month and 3-month)
- This is a univariable analysis to understand the effect of the mobile app intervention on each health and process outcome.
- The covariates are not able to be adjusted due to the low power of analysis as this study is a feasibility study involving a small sample size.
- The equation is stated below:

y= α +β (time point)+ ε

y is the health outcomes at time 0^th^ -month, 1-month and 3-month; α is the intercept; β is the coefficient of time point; ε is random effect assumed to follow a normal distribution with mean zero and variance.
