# Supplementary material for: A self-management app to improve asthma control in adults with limited health literacy: a mixed-method feasibility study
Source: BMC Med Inform Decis Mak. 2023 Sep 27;23:194. doi: 10.1186/s12911-023-02300-6 (PMC10523795; doi:10.1186/s12911-023-02300-6)
Supplement: Supplementary file 5 — Additional file 5. [file 12911_2023_2300_MOESM5_ESM.docx]

# Supplementary file 5
